# A MexR Mutation Which Confers Aztreonam Resistance to *Pseudomonas aeruginosa*

**DOI:** 10.3389/fmicb.2021.659808

**Published:** 2021-06-24

**Authors:** Zhenzhen Ma, Congjuan Xu, Xinxin Zhang, Dan Wang, Xiaolei Pan, Huimin Liu, Guangbo Zhu, Fang Bai, Zhihui Cheng, Weihui Wu, Yongxin Jin

**Affiliations:** ^1^State Key Laboratory of Medicinal Chemical Biology, Key Laboratory of Molecular Microbiology and Technology of the Ministry of Education, Department of Microbiology, College of Life Sciences, Nankai University, Tianjin, China; ^2^Tianjin Union Medical Center, Nankai University Affiliated Hospital, Tianjin, China

**Keywords:** *Pseudomonas aeruginosa*, aztreonam resistance, *mexR*, MexAB-OprM, mutation

## Abstract

Therapy for *Pseudomonas aeruginosa* infections is hard due to its high natural and acquirable antibiotic resistance. After colonization in the hosts, *P. aeruginosa* commonly accumulates genomic mutations which confer them antibiotic resistance and better adaptations to the host environment. Deciphering the mechanisms of antibiotic resistance development in the clinical setting may provide critical insights into the design of effective combinatory antibiotic therapies to treat *P. aeruginosa* infections. In this work, we demonstrate a resistance mechanism to aztreonam of a clinical isolate (ARP36) in comparison with a sensitive one (CSP18). RNAseq and genomic DNA resequencing were carried out to compare the global transcriptional profiles and in the clinical setting genomic profiles between these two isolates. The results demonstrated that hyperexpression of an efflux pump MexAB-OprM caused by a R70Q substitution in MexR, contributed to the increased resistance to aztreonam in the isolate ARP36. Simulation of *mexR* of ARP36 by gene editing in CSP18 conferred CSP18 an ARP36-like susceptibility to the aztreonam. The R70Q substitution prevented MexR from binding to the intergenic region between *mexR* and *mexAB-oprM* operon, with no impact on its dimerization. The presented experimental results explain for the first time why the clinically relevant R70Q substitution in the MexR derepresses the expression of *mexAB-oprM* in *P. aeruginosa.*

## Introduction

*Pseudomonas aeruginosa*, an opportunistic human pathogen, is a leading cause of both community-acquired and hospital-acquired infection all around the world (Driscoll et al., 2007). Infections by this bacterium are often difficult to cure because of its natural and acquirable resistance to a wide range of antibiotics, leaving a limited number of effective antimicrobial agents. The natural antibiotic resistance of *P. aeruginosa* is attributable to its low outer membrane permeability as well as to the expression of multidrug efflux pumps, such as MexAB-OprM (Nikaido, 1994; Li et al., 1995; Nakae, 1995). Of note, in addition to contributing to the natural resistance due to its constitutive expression, the MexAB-OprM is also hyperexpressed *via* mutations in *nalB*, *nalC*, or *nalD*, conferring multidrug-resistant phenotype (Saito et al., 1999; Srikumar et al., 2000; Adewoye et al., 2002; Cao et al., 2004; Sobel et al., 2005; Morita et al., 2006). Once infection is established, *P. aeruginosa* strains usually develop antibiotic resistance during antimicrobial treatment, and the same *P. aeruginosa* lineage typically persists in the patient (Mahenthiralingam et al., 1996; Burns et al., 2001).

Aztreonam is a fully synthetic β-lactam antibiotic, with a high binding affinity for penicillin-binding protein 3 (PBP3) and high stability against hydrolysis by a wide range of β-lactamases (Brogden and Heel, 1986). It has been approved in an inhaled formulation in treatment for cystic fibrosis patients with *P. aeruginosa* infection since 2010 (O’Sullivan et al., 2010). It has been shown that the inhaled aztreonam is an effective and safe antimicrobial drug to eradicate the newly acquired *P. aeruginosa* and for long-term suppressive treatment for chronic endobronchial infections in CF patients (Elson et al., 2019). However, as for all antimicrobial agents, *P. aeruginosa* can develop aztreonam resistance that decreases its effectiveness (Oermann et al., 2011). Although a few mechanisms of aztreonam resistance have been identified *via in vitro* evolution (Quale et al., 2006; Berrazeg et al., 2015; Jorth et al., 2017), understanding of the mechanisms for aztreonam resistance in the clinical setting is limited and needs to be further explored (McLean et al., 2019).

In this study, we obtained two clinical strains of *P. aeruginosa* from a patient with ulcerative colitis before and after therapy with cefoxitin. The early strain was isolated soon after the patient was admitted to the hospital, while the later one was isolated after the antibiotic therapy for 7 days. The early isolate CSP18 [named as CSP18 due to its sensitivity to ciprofloxacin in our earlier study (Xu et al., 2020a)]was susceptible to aztreonam, while the later one ARP36 was aztreonam resistant. Therefore, we wanted to explore the molecular mechanisms of aztreonam resistance in the resistant clinical isolate in comparison with the sensitive one in the clinical setting. The results presented in this study demonstrate that hyperexpression of the *mexAB-oprM* caused by a R70Q substitution in MexR is the contributory factor for this conversion. Furthermore, we have revealed that R70Q substitution in the MexR had no impact on its dimerization, rather it impaired its binding capability to the intergenic region between *mexR* and *mexAB-oprM* operon, thus derepressed the expression of *mexAB-oprM* operon. Our research provides further insights into the molecular mechanism of aztreonam resistance in *P. aeruginosa* under clinical setting.

## Materials and Methods

### Basic Characterization of the Bacterial Isolates

Bacterial strains and plasmids for this work are listed in Supplementary Table 1. Aztreonam-susceptible (CSP18) and aztreonam-resistant (ARP36) *P. aeruginosa* strains used in this work were isolated respectively from sputum and abdominal drainage of a patient with ulcerative colitis before and after 7-day therapy with cefoxitin, which was used as an empirical anti-infective drug. Species of these two isolates were identified by PCR amplification with primers (Supplementary Table 2) and sequence analysis of 16S rDNA (Spilker et al., 2004). Random amplified polymorphic DNA (RAPD) typing was conducted with indicated primer (Supplementary Table 2) according to a previous study (Mahenthiralingam et al., 1996). The allelic profiles of the *P. aeruginosa* isolates were determined by multilocus sequence typing (MLST) analysis following previous description (Curran et al., 2004). Briefly, chromosomal DNAs were isolated from overnight cultures of *P. aeruginosa* strains using DNA purification kit (Tiangen Biotec, Beijing, China) and utilized as PCR templates. The internal fragments of *aroE*, *mutL*, *acsA*, *guaA*, *nuoD*, *trpE*, and *ppsA* genes were amplified with PCR, sequenced with indicated primers (shown in Supplementary Table 2), and the sequences were then submitted to the *P. aeruginosa* MLST database^1^ to obtain the allelic numbers. A sequence type (ST) was assigned to CSP18 or ARP36 by combination of the seven allelic numbers. Minimum inhibitory concentration (MIC) against antibiotics was tested by a twofold serial dilution method described before with the exception that the bacteria were cultured in LB broth (Richardot et al., 2016), and susceptibility to aztreonam was interpreted based on the Clinical and Laboratory Standards Institute guidelines (CLSI 2011–2018).

### Construction of Plasmids

To overexpress *mexR*, a 495 bp *mexR*-containing fragment was amplified with PCR using CSP18 and ARP36 genomic DNA as templates (primers listed in Supplementary Table 2). The resulting PCR products were digested with *Eco*RI and *Bam*HI, and then inserted into pUCP24, leading to pUCP24-*mexR*_CSP18_ and pUCP24-*mexR*_ARP36_, respectively. For the expression of C-terminal His-tagged MexR in *Escherichia coli*, the open reading frame of *mexR* without its stop codon was amplified with PCR using CSP18 and ARP36 genomic DNA as templates (primers listed in Supplementary Table 2). The resulting PCR products were digested with *Xho*I and *Bam*HI, and then inserted into an expression vector pET28a, leading to pET28a-*mexR*_CSP18_ and pET28a-*mexR*_ARP36_, respectively. For bacterial two-hybrid assay, the *mexR* gene of CSP18 and ARP36 were amplified with the indicated primers (Supplementary Table 2) and respectively cloned into the prey vector (pTRG) and bait vector (pBT).

To make the *mexR* (MexR R70Q) point mutation construct, a 1,135 bp DNA fragment containing *mexR* and partial immediately adjacent genes (*pasP and mexA*) was PCR amplified using ARP36 genomic DNA as templates (primers listed in Supplementary Table 2), digested with *Bam*HI and *Eco*RI, and then inserted into a pEX18Tc vector, resulting in pEX18-*mexR*_ARP36_. Gene editing in CSP18 was carried out by conjugal transfer of this plasmid followed by selection for single crossover and then double crossover, as early described (Schweizer, 1992). The target strain with MexR R70Q point mutation was confirmed by PCR amplification and sequencing analysis (primers in Supplementary Table 2).

### Total RNA Isolation, RT-qPCR, and RNAseq Analysis

Total RNA isolation and RT-qPCR were carried out as described in our early studies (Xu et al., 2020a; Xu et al., 2020b). Overnight bacterial strains were subcultured to the log phase (OD_600_ = 1.0) at 37°C. Total RNA was extracted using RNAprep Pure Cell/Bacteria Kit (Tiangen Biotec, Beijing, China) and dissolved into RNase free water. cDNAs were synthesized with random primers and PrimeScript Reverse Transcriptase (Takara, Dalian, China) and mixed with specific primers (Supplementary Table 2) and SYBR premix Ex Taq II (Takara, Dalian, China). Quantitative PCR was carried out in a CFX Connect Real-Time Machine (Bio-Rad, Hercules, CA, United States). As an internal control, *rpsL*, encoding a 30S ribosomal protein was utilized.

RNAseq was performed as described before (Xu et al., 2020a,b). The quality and quantity of total RNAs from CSP18 and ARP36 were determined with a Bioanalyzer 2100 system (Agilent Technologies, Palo Alto, CA, United States), a NanoDrop spectrophotometer, and electrophoresis on a 1% (wt/vol) agarose gel. A Ribo-Zero rRNA Removal Kit (Bacteria, Illumina, San Diego, CA, United States) was used to remove rRNA. After that, mRNA was fragmented and reverse transcribed to synthesize the double-strand cDNA, followed by purification, ends repair, and ligation with adaptors. After 11 cycle PCR reaction, the products were purified, qualified, and quantified using a Bioanalyzer 2100 system (Agilent), and a Qubit 2.0 Fluorometer (Invitrogen, Carlsbad, CA, United States). The resulting DNA libraries were then sequenced using an HiSeq 2500 platform (Illumina) with a 2 × 150 paired-end configuration.

Sequencing reads were mapped onto the reference genome of PAO1 (NC_002516.2) with Bowtie2 (v2.1.9). Gene expressional values were then analyzed by software HTSeq (v0.6.1p1). Differentially expressed genes were identified with the DESeq software, with a cut-off of fold-change more than 2 and *p*-value no larger than 0.05.

### DNA Purification and Resequencing

DNA purification and resequencing were conducted as previously described (Xu et al., 2020a,b). Genomic DNA of bacterial strains was extracted with DNA isolation kit (Tiangen Biotec, Beijing, China). Fragments shorter than 500 bp in length were recovered from 200 ng chromosomal DNA using sonication (Covaris S220), ends treated, and then ligated with an adaptor. The fragments ∼470 bp were purified with beads and then amplified in a six-cycle PCR reaction. The products were purified with beads, qualified, and quantified with a Qsep100 machine (Bioptic, Taiwan, China) and a Qubit3.0 Fluorometer (Invitrogen). The generating libraries were sequenced on a Hiseq machine (Illumina) with a 2 × 150 paired-end (PE) configuration following manufacturer’s instructions (Illumina). The sequencing reads were mapped onto the reference genome of PAO1 (NC_002516.2) via a BWA software (version 0.7.12). Single nucleotide variation (SNV) and InDel mutation were analyzed with the software Samtools (version 1.1) and the Unified Genotyper module from GATK.

### Expression and Purification of MexR-His

MexR-His recombinant protein was purified as previously described with a minor modification (Evans et al., 2001). Briefly, an overnight culture of *E. coli* BL21/DE3 containing plasmid pET28a-*mexR*_CSP18_ or pET28a-*mexR*_ARP36_ was diluted 50-fold into 100 ml of L-broth supplemented with kanamycin (50 μg/ml) and grown at 37°C until the culture reached an OD_600_ of 0.4–0.6. Expression of MexR-His was then induced by adding isopropyl-β-_*D*_-thiogalactoside (IPTG) at a final concentration of 0.1 mM and growing for another 4 h. The bacterial cells were collected by centrifugation at 8,000 × g for 10 min at 4°C and resuspended in the lysis buffer (50 mM Na_2_HPO_4_, 50 mM NaH_2_PO_4_, 0.3 M NaCl), followed by sonication until the suspension became limpid. The recombinant MexR-His was Ni-affinity purified from soluble fraction following the manufacturer’s instruction (Qiagen). The purified protein was eluted with lysis buffer containing 300 mM imidazole, examined by SDS-PAGE, quantified by Nanodrop, and stored at 4°C.

### Electrophoretic Mobility Shift Assay

Electrophoretic mobility shift assay (EMSA) was carried out as described before with minor modification (Zheng et al., 2018). Briefly, DNA fragments of the intergenic region of *mexA-mexR* were PCR amplified with indicated primers (Supplementary Table 2). DNA fragments (0.35 μM) were incubated with purified MexR-His protein (0–36 μM) in a 20 μl reaction system (50 mM Tris, 0.5 M EDTA, 50 mM NaCl, 2.5% glycerol, pH 7.9) at 37°C for 30 min. Samples were loaded onto an 8% native polyacrylamide gel in 0.5 × Tris-Borate-EDTA (TBE) buffer which had been prerun on ice for 1 h and electrophoresed on ice for 1.5 h at 120 V. The gel was then stained with 0.5 μg/ml ethidium bromide in a 0.5 × TBE buffer and visualized in an imager ChemiDocTM XRS + (Bio-Rad).

### Microscale Thermophoresis Analysis

The microscale thermophoresis analysis (MST) experiments were conducted on Monolith NT.115 instrument (NanoTemper, München, Germany) according to the manufacture’s instruction. A DNA fragment corresponding to the intergenic region of *mexR-mexA* was PCR amplified with primers shown in Supplementary Table 2. The proteins MexR_CSP18_ and MexR_ARP36_ were labeled with NT-647-NHS as aptamer probes. Samples containing 352 nM labeled MexR_CSP18_ or MexR_ARP36_ and serial twofold increasing concentrations of DNA fragments (0.488–16,000 nM) in binding buffer (10 mM Tris, 1 mM DTT) were loaded on standard treated silicon capillaries (NanoTemper Technologies). Thermophoresis was measured with a Monolith NT.115 instrument at room temperature using 20% light-emitting diode (LED) and 60% MST power. Data were analyzed using MO affinity analysis software.

### Other Methods

The β-galactosidase activity was measured as described previously (Weng et al., 2016). Assays for protein-protein interaction by bacterial two-hybrid system were performed according to the protocol supplied by the manufacturer (Stratagene, La Jolla, CA, United States).

### Statistical Analysis

Statistical analyses were performed using Graphpad software. Student’s *t*-test (two-tailed) was utilized to assess the statistical significance of two-group comparisons.

### Ethics Statement

We have a waiver from the medical ethics committee of Tianjin Union Medical Center, exempting this study from the requirement to have ethics approval and written informed consent as the clinical strains used in this work come from the routine procedures of the clinical laboratory rather than the clinical trials.

## Results and Discussion

### Clinical Isolates CSP18 and ARP36 Belong to the Same Clone

**FIGURE 1:**
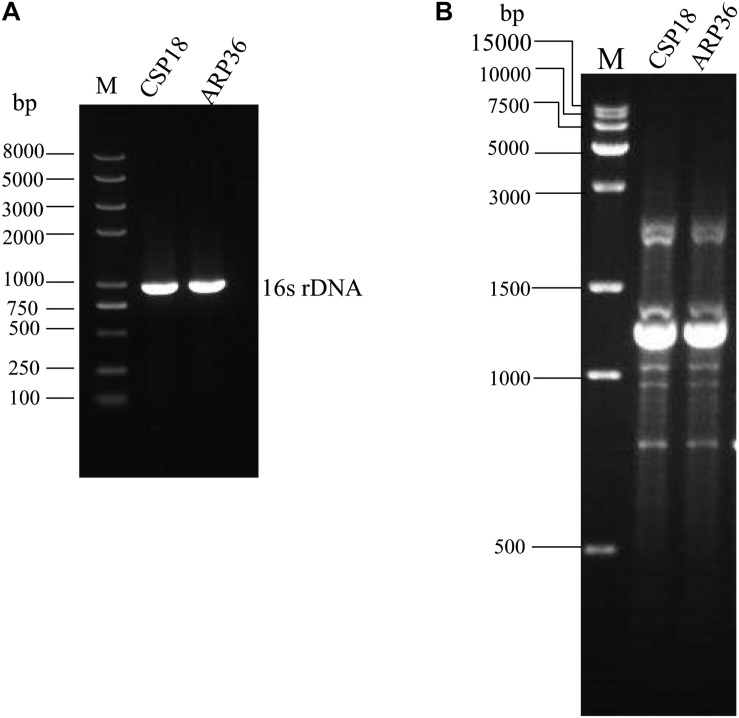
PCR results of CSP18 and ARP36 strains. **(A)** 16S rDNA gene amplification. **(B)** RAPD typing.

Sputum and abdominal drainage samples were collected from a patient with ulcerative colitis before and after therapy for 7 days with cefoxitin. Two *P. aeruginosa* strains were isolated, the early one before the antibiotic therapy which was susceptible to aztreonam (CSP18), and the later one after the antibiotic therapy which was resistant against aztreonam (ARP36) (Table 1). PCR examination and sequencing analysis for the 16S rDNAs revealed that both of them were *P. aeruginosa* (Figure 1A) (Spilker et al., 2004). The allelic profile of *guaA*, *mutL*, *nuoD*, *acsA*, *aroE*, *ppsA*, and *trpE* genes were revealed as 12, 98, 4, 17, 5, 14, and 10, respectively by MLST analysis, representing the same sequence type, ST611. A RAPD typing was further conducted on the CSP18 and ARP36 strains, which in combination with MLST analysis indicated that they belonged to the same clone (Figure 1B).

**TABLE 1:** MICs (μg/ml) of indicated *P. aeruginosa* strains.

Strains	Azt^a,b^	Caz^b^	Mem^b^	Fep^b^	Pip^b^	Chl^b^	Cip^b^	Ofl^b^	Fox^b^
CSP18	4	1	0.5	1	16	64	0.25	1	2,048
ARP36	32	4	2	4	64	256	1	4	2,048
CSP18/pUCP24	4	1	0.5	1	16	64	0.25	1	2,048
ARP36/pUCP24	32	4	2	4	64	256	1	4	2,048
CSP18/*mexR*_CSP18_	4	1	0.5	1	16	64	0.25	1	2,048
CSP18/*mexR*_ARP36_	8	2	1	2	32	128	0.5	2	2,048
ARP36/*mexR*_CSP18_	16	2	1	2	32	128	0.5	2	2,048
ARP36/*mexR*_ARP36_	32	4	2	4	64	256	1	4	2,048
CSP18*mexR*_ARP36_	32	4	2	4	64	256	1	4	2,048

**^*a*^Clinical Laboratory Standards Institute (CLSI) susceptibility breakpoints: aztreonam ≤ 8 μg/ml; resistance breakpoints: aztreonam ≥ 32 μg/ml.*^*b*^*Azt, aztreonam; Caz, ceftazidime; Mem, meropenem; Fep, cefepime; Pip, piperacillin; Chl, chloramphenicol; Cip, ciprofloxacin; Ofl, ofloxacin; Fox, cefoxitin.**

### Isolate ARP36 Is Resistant to Aztreonam

Aztreonam is an effective anti-*P. aeruginosa* drug (Elson et al., 2019), and aztreonam susceptibility of CSP18 and ARP36 were different based on the preliminary examination with VITEK automatic microbe analysis instrument (data not shown). Therefore, their susceptibilities to aztreonam were further examined with the twofold serial dilution method (Richardot et al., 2016). As shown in Table 1, CSP18 (the early isolate) was found to be susceptible to aztreonam, with MIC of 4 μg/ml, while the later one (ARP36) displayed resistance to aztreonam, with MIC of 32 μg/ml, representing an eightfold increase (Table 1).

### An Operon Encoding MexAB-OprM Efflux Pump Is Upregulated in ARP36

**FIGURE 2:**
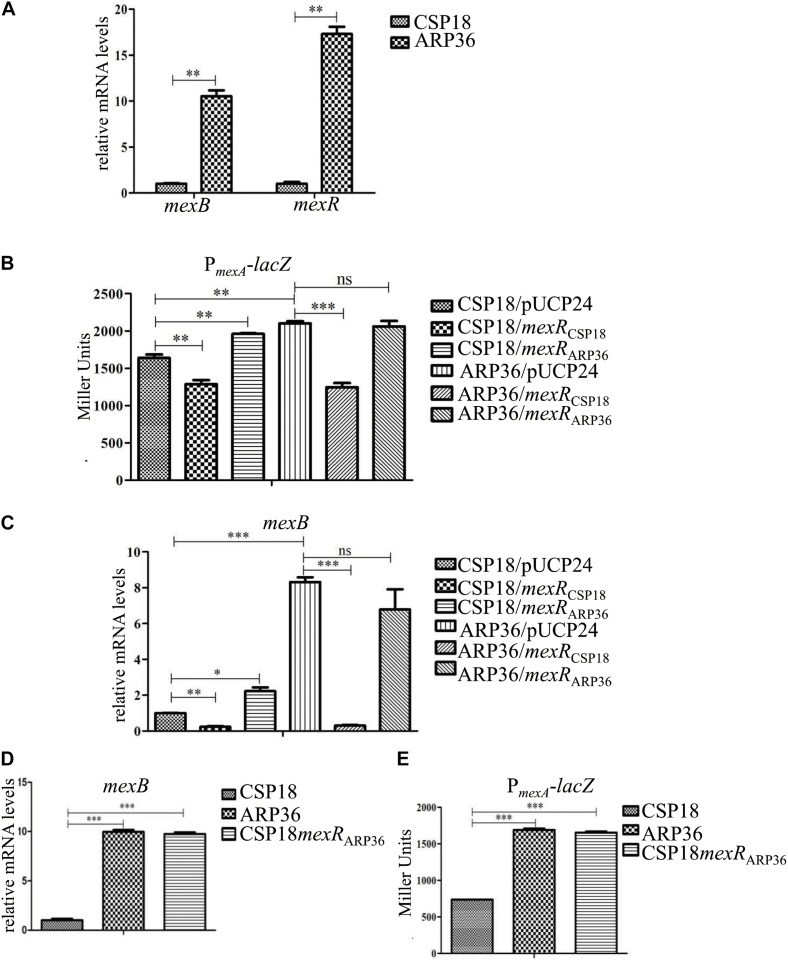
Transcriptional levels of indicated genes in indicated strains. **(A)** Relative mRNA levels of *mexB* **(A**,**C**,**D)** and *mexR* **(A)** in indicated strains. Total RNA was isolated from indicated strains at OD_600_ of 1.0, and the relative mRNA levels of indicated genes were examined by real-time qPCR with *rpsL* as an internal control. **(B,E)** β-galactosidase assay was carried out to examine the transcriptional activity of *mexAB-oprM* operon promoter fused with a *lacZ* gene in indicated strains. ns, not significant; *P < 0.05; **P < 0.01; ***P < 0.001, by Student’s *t*-test.

To explore the mechanisms of reduced susceptibility to aztreonam in the isolate ARP36, RNAseq was performed and the global gene expression profiles were compared between the strains CSP18 and ARP36. mRNA levels of 30 genes were significantly altered between these two isolates (Supplementary Table 3). Among them, genes encoding efflux pump MexAB-OprM, *mexA*, *mexB*, and *oprM*, displayed eight-, seven-, and sevenfold higher mRNA levels, respectively, in ARP36 than those in CSP18 (Supplementary Table 3). To validate the increased transcriptional level, we further examined and compared the mRNA levels of *mexAB-oprM* operon between CSP18 and ARP36 by real-time qPCR. Consistent with the RNAseq results, the mRNA level of *mexB* showed a significant increase in ARP36 compared with that in CSP18 (Figure 2A). To further confirm the increased expression level of the *mexAB-oprM*, P*_*mexA*_*-*lacZ* reporter construct was transformed into the CSP18 and ARP36 strains and their β-galactosidase activities were measured. As shown in Figures 2B,E, the ARP36 strain displayed an increased β-galactosidase activity, which further conformed with the RNAseq and real-time qPCR results. In addition, the transcriptional level of *mexR*, encoding a repressor for the *mexAB-oprM* operon (Poole et al., 1996), displayed a 15-fold increase in the ARP36 strain in comparison with the CSP18 strain in the RNAseq results (Supplementary Table 3). The increased mRNA level of *mexR* was further confirmed by the real-time qPCR (Figure 2A).

It has been reported that MexAB-OprM efflux pump can export a large number of antibiotics belonging to different classes, including β-lactams, fluoroquinolones, and chloramphenicol (Lister et al., 2009). Therefore, we also examined the sensitivity of CSP18 and ARP36 against ceftazidime, meropenem, piperacillin, cefepime, chloramphenicol, ciprofloxacin, and ofloxacin. Similar to aztreonam, ARP36 showed reduced susceptibility to all of these antibiotics, with an average fourfold increase in MIC against them (Table 1). However, the ARP36 displayed a similar susceptibility to cefoxitin as the CSP18 (Table 1), which suggested that cefoxitin may not be a substrate for the MexAB-OprM efflux pump. Of note, while cefoxitin is not an antipseudomonal antibiotic, it has been reported to induce expression of *ampC* strongly in *P. aeruginosa* (Livermore et al., 2017). However, the selective pressure of cefoxitin for the aztreonam resistance development remains elusive. In addition, other factors, such as the inflammatory response, nutrient availability, and oxygen levels in the ulcer location of the host may also contribute to this development.

### An Amino Acid Substitution in MexR Contributes to the Upregulation of *mexAB-oprM* and Increased Resistance to Aztreonam in ARP36

To further explore the molecular mechanism for the increased resistance against aztreonam in ARP36, as well as the increased expression of the *mexAB-oprM* operon, DNA resequencing was conducted to determine mutations in the genome of ARP36 relative to that of the CSP18 strain, with PAO1 as reference genome^2^. Between CSP18 and ARP36, there were 207 variants, among which 76 variants were heterozygous (Supplementary Table 4), suggesting they may occur during the culture *in vitro* before the DNA extraction. According to the DNA resequencing results, 28 genes had insertions or deletions, while 16 genes had non-synonymous single nucleotide variation (SNV) (Supplementary Table 4). Among them, a G209A substitution in the *mexR* gene resulted in a R70Q substitution in the MexR protein of the ARP36 strain in comparison with that of CSP18 (Supplementary Table 4). To validate the mutation, *mexR* genes were amplified by PCR from the genomic DNA of strains CSP18 and ARP36 and cloned into pUCP24 vectors. The *mexR* clones were sequenced and aligned with the *mexR* gene of PAO1 reference strain^2^. Sequence analysis revealed that *mexR* in CSP18 was the same as that of PAO1 reference^2^, while *mexR* from ARP36 showed a G209A substitution, which confirmed the results of genome resequencing.

MexR, a MarR family transcriptional regulator, is known to repress the expression of *mexAB-oprM* and itself in *P. aeruginosa* (Poole et al., 1996). Mutations in the MexR lead to *mexAB-oprM* hyperproduction, and an attendant increased antibiotics resistance had been reported previously (Ziha-Zarifi et al., 1999; Saito et al., 2003; Choudhury et al., 2016). To assess if the R70Q substitution in MexR contributed to the increased expression of *mexAB-oprM* operon as well as the decreased susceptibility to aztreonam in the ARP36 strain, the *mexR* gene from both CSP18 and ARP36 were overexpressed in the ARP36 strain background. The mRNA levels of *mexB* were compared by real-time qPCR. As the results shown in Figure 2C, introduction of the *mexR*_CSP18_, but not *mexR*_ARP36_, decreased the relative mRNA levels of *mexB*_ARP36_. Also, P*_*mexA*_*-*lacZ* reporter assay further confirmed these results (Figure 2B). Not totally consistent with the reduced expression of the *mexB*, the MIC against aztreonam of ARP36 containing *mexR*_CSP18_ was decreased only twofold (Table 1), not to the level of CSP18. Plasmids expressing the *mexR*_CSP18_ or *mexR*_ARP36_ were further respectively introduced into the CSP18 strain background. As the results shown in Figures 2B,C and Table 1, overexpression of *mexR*_ARP36_, but not *mexR*_CSP18_, resulted in significant increases in β-galactosidase activity, *mexB* mRNA level, and MIC against aztreonam. These data demonstrated that the R70Q substitution in MexR is the cause of dramatic increase in the expression of *mexAB-oprM* seen in the ARP36 strain.

The fact that wild-type *mexR* (*mexR*_CSP18_) was unable to restore the MIC against aztreonam in ARP36 to the level of CSP18 suggested that either some other factors may also be involved or the MexR_ARP36_ functions dominant over MexR_CSP18_. To test this possibility, the chromosomal *mexR* gene of the CSP18 strain was replaced by *mexR*_ARP36_. As shown in Table 1, replacement of the *mexR*_CSP18_ with *mexR*_ARP36_ conferred the CSP18 strain the same MIC against aztreonam as the ARP36 strain. Agreeing with the recovered MIC to aztreonam of CSP18*mexR*_ARP36_, transcriptional level of *mexAB-oprM* was also increased to that of ARP36 (Figures 2D,E). These data demonstrated that R70Q substitution in the MexR is responsible for the reduced susceptibility to aztreonam in strain ARP36 and further indicated that the MexR (R70Q) encoded by the ARP36 displays a dominant negative effect over the functional MexR_CSP18_.

### R70Q Substitution Prevents MexR From Binding to the Intergenic Region Between *mexR* and *mexAB-oprM* Operon

**FIGURE 3:**
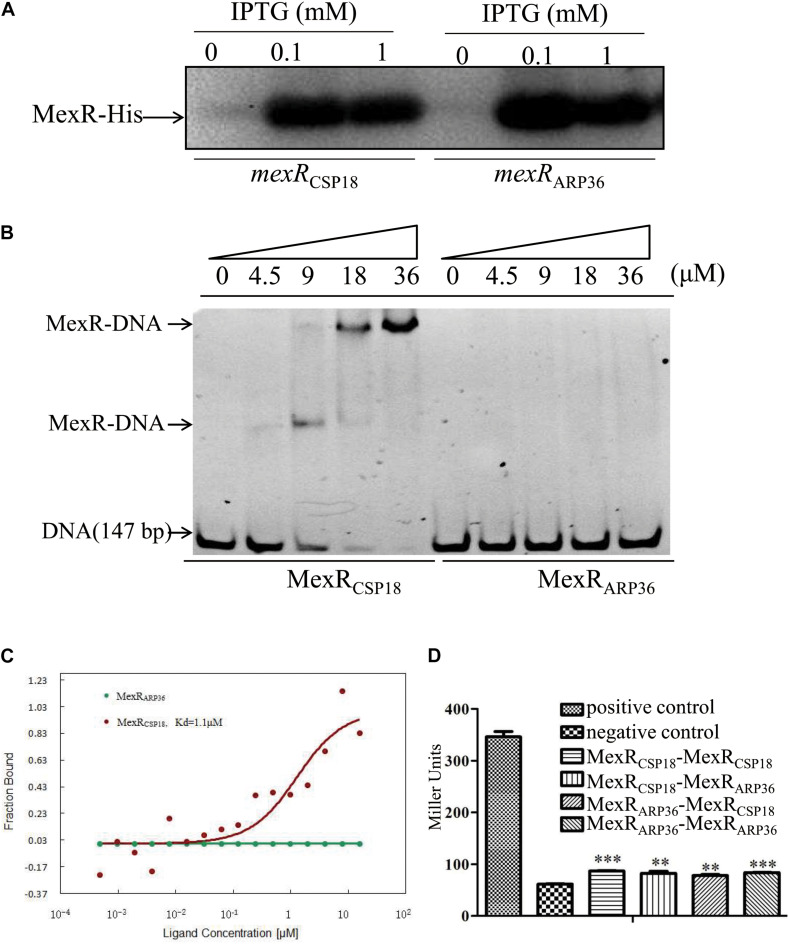
Binding of MexR_CSP18_ or MexR_ARP36_ to the intergenic region of *mexR*-*mexAB-oprM* and interaction between MexR_CSP18_ and MexR_ARP36_. **(A)** MexR-His protein detected by Western blot. **(B)** Binding of MexR to its target DNA was determined by EMSA. Increasing amount of the purified MexR_CSP18_-His or MexR_ARP36_-His protein was incubated with intergenic region of *mexR*-*mexA*. The mixtures were electrophoresed on a native PAGE gel, and the bands were visualized under UV light following ethidium bromide staining. Data represent results from three independent experiments. **(C)** MST assay to test the binding capability of MexR_ARP36_ or MexR_CSP18_ to the intergenic region of *mexR*-*mexA*. **(D)** Test of protein-protein interactions between MexR_CSP18_ and MexR_ARP36_ using the bacterial-matched two-hybrid system. **P < 0.01; ***P < 0.001, by Student’s *t*-test.

MexR binds as a dimer to the intergenic region between *mexR* and *mexAB-oprM* operon to repress their expressions (Evans et al., 2001; Adewoye et al., 2002). The R70Q substitution in MexR resulted in a dramatic increase in the expression of *mexAB-oprM* in the ARP36 strain. This prompted us to test the binding capability of MexR (R70Q) to the target sequence. EMSA was performed using a fragment between *mexR* and *mexAB-oprM* operon which contains two MexR-binding sites (Evans et al., 2001). MexR_CSP18_ and MexR_ARP36_ were expressed in *E. coli* BL21 (DE3), purified with Ni-NTA (Figure 3A), and used for EMSA. As shown in Figure 3B, band shift was detected when DNA fragments were incubated with MexR_CSP18_, but not MexR_ARP36_, indicating that MexR_APR36_ lost its binding capability to the target sequence. To further confirm this result, MST experiment was carried out with NT-647-NHS-labeled MexR as aptamer probes. As the results shown in Figure 3C, the binding between MexR_CSP18_ and *mexR-mexA* intergenic region was detected with a *K*_*d*_ of 1.1 μM, while the binding of MexR_ARP36_ was undetectable. These results further confirmed that MexR with a R70Q substitution compromised its binding capability to the *mexR-mexA* intergenic region. Notably, some other mutations in MexR, such as L57P, L57R, T69I, etc., also render MexR unable to bind to its DNA binding region (Saito et al., 2003).

As MexR forms homodimer for DNA binding (Evans et al., 2001; Lim et al., 2002), we want to ask if the impaired binding capability of MexR_ARP36_ was due to the abolished dimerization. To assess the possibility, we utilized a bacterial two-hybrid system to assess MexR dimerization. The *mexR* genes from CSP18 and ARP36 were respectively cloned into both the pTRG and pBT vectors. The interactions between each two protein pairs were assessed by the β-galactosidase activity in the reporter strains. Test results showed positive interaction between MexR_ARP36_ and MexR_ARP36_ or MexR_CSP18_ and MexR_ARP36_, indistinguishable with that between MexR_CSP18_ and MexR_CSP18_ (Figure 3D). These data demonstrated that R70Q substitution had no apparent impact on the dimerization of MexR and provided a possible explanation for the dominance of MexR_ARP36_ over MexR_CSP18_. Of note, L95F or R21W substitution in MexR had been shown to have no apparent impact on MexR dimerization and be dominant over the wild-type MexR when coexpressed in *P. aeruginosa* (Adewoye et al., 2002).

In fact, a R70W mutation in MexR in *nalB* strain OCR1 has been reported to lead to overexpression of *mexAB-oprM* (Poole et al., 1996). Similar to many other *nalB* strains, OCR1 strain produced little or no detectable MexR protein (Adewoye et al., 2002). Similarly, a R73C substitution in MarR, another member of MarR family regulator, resulted in a low protein amount when expressed in *E. coli* BL21 (DE3) (Alekshun et al., 2000). However, in our study, similar MexR protein amounts were observed between MexR_CSP18_ (MexR_WT_) and MexR_ARP36_ (MexR_R70Q_) when expressed in *E. coli* BL21 (DE3) (Figure 3A), indicating the different effect on MexR stability mediated by MexR_R70Q_ and MexR_R70W_.

**FIGURE 4:**
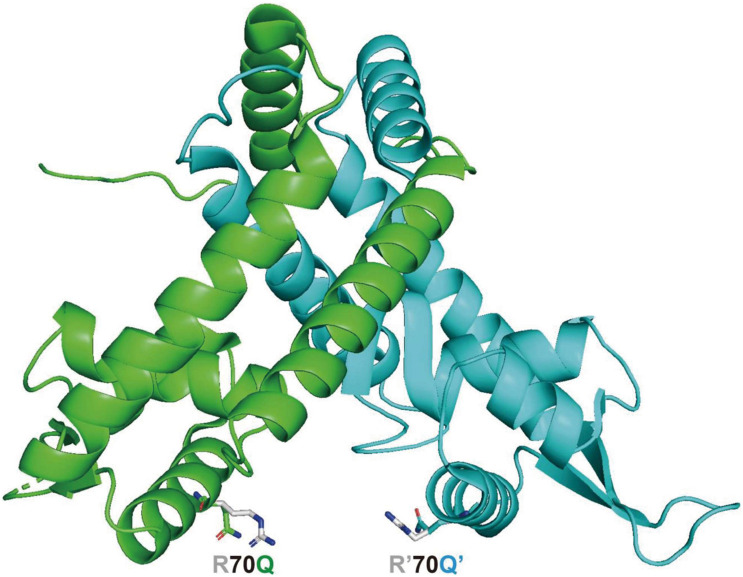
Model structure of MexR R70Q protein in the open (able to bind DNA) form, based on the “Crystal structure of the MexR repressor of the *mexRAB-oprM* multidrug efflux operon of *Pseudomonas aeruginosa*” (Lim et al., 2002) (PDB entry 1LNW), side chain of the mutant residue was generated by PyMOL.

The three-dimensional structure of MexR and MarR has been solved with X-ray crystallography (Lim et al., 2002; Wilkinson and Grove, 2006). The critical biophysical properties of MexR and its well-established set of *mexR* mutations, including R70W substitution, have been analyzed (Andrésen et al., 2010). MexR lacks tryptophan, while has 12 arginines. In addition, R70W and R70Q substituted MexR exerted different effects on the MexR stability. Therefore, the three-dimensional structure of R70Q mutant of MexR was further modeled based on the crystal structure of the MexR in its open form (PDB entry 1LNW) (Lim et al., 2002), as shown in Figure 4. It is suggested that this mutation on alpha helix4, or the DNA recognition helix, could have profound effect on disrupting the DNA-binding conformation in two possible manners: (i) the mutant may break the electrostatic interaction formed by the originally positively charged Arg70 and the phosphate group of the binding DNA; (ii) this mutation may also alter the conformation of α helix4 on two protomers of the MexR dimer, and consequently lead to a narrower groove which is incompatible with DNA binding. However, this mutant may not significantly alter the dimeric assembly as α helix4 has not been shown as a structural element for dimerization. To further explore the molecular basis for the changed regulation mode as observed in this study, the crystal structure of the R70Q mutant should be determined.

There are numerous regulators that have been shown to influence the expression of *mexAB-oprM* operon genes. In addition to the MexR, NalD, a second repressor, represses the expression of *mexAB-oprM* directly by binding to its second promoter (Morita et al., 2006). NalC, a repressor of *armR*, regulates *mexAB-oprM* expression through repressing the expression of ArmR, which can bind to and prevent MexR binding to the promoter region of *mexAB-oprM* (Cao et al., 2004). CpxR, a two-component response regulator, and BrlR, a MerR-like regulator, positively regulate *mexAB-oprM* expression by directly binding to its promoter region (Liao et al., 2013; Tian et al., 2016). MdrR1 (PA3898) and MdrR2 (PA2100) directly repress the expression of *mexAB-oprM* multidrug efflux pump independent of the MexR (Heacock-Kang et al., 2018). MexT, a LysR-type regulator, and RocA2, a two-component system regulator, exert a negative regulatory effect on *mexAB-oprM* genes (Maseda et al., 2004; Sivaneson et al., 2011). However, all the genes mentioned above are identical between CSP18 and ARP36 (data not shown), thus were not the cause for the increased expression of *mexAB-oprM* in ARP36.

## Data Availability Statement

The raw data for both RNAseq and genome resequencing have been upload to the NCBI, under the accession number: PRJNA681301. And the data can be found under the link below: https://www.ncbi.nlm.nih.gov/sra/?term=PRJNA681301.

## Author Contributions

YJ conceived, designed the experiments, and wrote the manuscript. ZM, CX, XZ, DW, XP, HL, and GZ performed the experiments. YJ, FB, ZC, and WW analyzed the data. All authors contributed to the article and approved the submitted version.

## Conflict of Interest

The authors declare that the research was conducted in the absence of any commercial or financial relationships that could be construed as a potential conflict of interest.

## Footnotes

1 http://pubmlst.org/paeruginosa/

2 http://www.pseudomonas.com

## Supplementary Material

Supplementary Table 1 Bacterial strains and plasmids used in this study.

Supplementary Table 2 Primers used in this study.

Supplementary Table 3 Transcriptome analysis: differentially expressed genes by RNAseq.

Supplementary Table 4 Genomic mutations identified by genome resequencing with elimination of SNVs with synonymous mutation and the same mutations between CSP18 and ARP36.
