# Supplementary material for: A MexR Mutation Which Confers Aztreonam Resistance to Pseudomonas aeruginosa
Source: Front Microbiol. 2021 Jun 24;12:659808. doi: 10.3389/fmicb.2021.659808 (PMC8264304; doi:10.3389/fmicb.2021.659808)
Supplement: Supplementary Table 1 — Bacterial strains and plasmids used in this study. [file Table_1.docx]

Table S1. Strains and plasmids used in this study

| Strains/plasmids | Description | Source/reference  (reference) |
| --- | --- | --- |
| Strains |  |  |
| CSP18 | an aztreonam sensitive clinical isolate | This study |
| ARP36 | an aztreonam resistant clinical isolate | This study |
| CSP18/pUCP24 | CSP18 containing pUCP24 | This study |
| CSP18/*mexR*_CSP18_ | CSP18 containing pUCP24-*mexR*_CSP18_ | This study |
| CSP18/*mexR*_ARP36_ | CSP18 containing pUCP24-*mexR*_ARP36_ | This study |
| ARP36/pUCP24 | ARP36 containing pUCP24 | This study |
| ARP36/*mexR*_CSP18_ | ARP36 containing pUCP24-*mexR*_CSP18_ | This study |
| ARP36/*mexR*_ARP36_ | ARP36 containing pUCP24-*mexR*_ARP36_ | This study |
| BL21/pET28a-*mexR*_CSP18_ | BL21 containing pET28a-*mexR*_CSP18_ | This study |
| BL21/pET28a-*mexR*_ARP36_ | BL21 containing pET28a-*mexR*_ARP36_ | This study |
| CSP18*mexR*_ARP36_ | CSP18 with native *mexR* replaced by *mexR*_ARP36_ | This study |
| RS  Plasmids | Bacterial two-hybrid reporter strain | Stratagene |
| Two-hybrid positive strain | Positive control for bacterial two-hybrid | Stratagene |
| Two-hybrid negative strain | Negative control for bacterial two-hybrid | Stratagene |
| Plasmids |  |  |
| pUCP24 | Broad-host-range shuttle vector; Gm^r^ | [[1](#_ENREF_1)] |
| pUCP24-*mexR*_CSP18_ | *mexR* from CSP18 cloned into pUCP24; Amp^r^ | This study |
| pUCP24-*mexR*_ARP36_ | *mexR* from ARP36 cloned into pUCP24; Amp^r^ | This study |
| pET28a-*mexR*_CSP18_ | *mexR* from CSP18 cloned into pET28a | This study |
| pET28a-*mexR*_ARP36_ | *mexR* from ARP36 cloned into pET28a | This study |
| pEX18Tc | Gene replacement vector; Tc^r^, oriT^+^, sacB^+^ | [[2](#_ENREF_2)] |
| pEX18-*mexR*_ARP36_ | *mexR* R70Q mutation construct in pEX18Tc, Tc^r^ | This study |
| pBT | Vector for bacteria two-hybrid, Ch1^r^ | Stratagene |
| pBT-*mexR*_CSP18_ | *mexR* from CSP18 cloned into pBT, Ch1^r^ | This study |
| pBT-*mexR*_ARP36_ | *mexR* from ARP36 cloned into pBT, Ch1^r^ | This study |
| pTRG | Vector for bacteria two-hybrid, Tc^r^ | Stratagene |
| pTRG-*mexR*_CSP18_ | *mexR* from CSP18 cloned into pTRG, Tc^r^ | This study |
| pTRG-*mexR*_ARP36_ | *mexR* from ARP36 cloned into pTRG, Tc^r^ | This study |

1. West, S.E., et al., *Construction of improved Escherichia-Pseudomonas shuttle vectors derived from pUC18/19 and sequence of the region required for their replication in Pseudomonas aeruginosa.* Gene, 1994. **148**(1): p. 81-6.

2. Hoang, T.T., et al., *A broad-host-range Flp-FRT recombination system for site-specific excision of chromosomally-located DNA sequences: application for isolation of unmarked Pseudomonas aeruginosa mutants.* Gene, 1998. **212**(1): p. 77-86.
