# Supplementary material for: A MexR Mutation Which Confers Aztreonam Resistance to Pseudomonas aeruginosa
Source: Front Microbiol. 2021 Jun 24;12:659808. doi: 10.3389/fmicb.2021.659808 (PMC8264304; doi:10.3389/fmicb.2021.659808)
Supplement: Supplementary Table 2 — Primers used in this study. [file Table_2.DOCX]

Table S2. Primers used in this study

| Primers | Sequence (5’→3’) | Use | Reference/Source |
| --- | --- | --- | --- |
| *acsA*-F | ACCTGGTGTACGCCTCGCTGAC | Gene amplification for MLST analysis | Curran *et al* (2004) |
| *acsA*-R | GACATAGATGCCCTGCCCCTTGAT |  |  |
| *aroE*-F | TGGGGCTATGACTGGAAACC |  |  |
| *aroE*-R | TAACCCGGTTTTGTGATTCCTACA |  |  |
| *guaA*-F | CGGCCTCGACGTGTGGATGA |  |  |
| *guaA*-R | GAACGCCTGGCTGGTCTTGTGGTA |  |  |
| *mutL*-F | CCAGATCGCCGCCGGTGAGGTG |  |  |
| *mutL*-R | CAGGGTGCCATAGAGGAAGTC |  |  |
| *nuoD*-F | ACCGCCACCCGTACTG |  |  |
| *nuoD*-R | TCTCGCCCATCTTGACCA |  |  |
| *ppsA*-F | GGTCGCTCGGTCAAGGTAGTGG |  |  |
| *ppsA*-R | GGGTTCTCTTCTTCCGGCTCGTAG |  |  |
| *trpE*-F | GCGGCCCAGGGTCGTGAG |  |  |
| *trpE*-R | CCCGGCGCTTGTTGATGGTT |  |  |
| 16s rDNAF | GGGGGATCTTCGGACCTCA | 16s rDNA PCR/sequencing | Spilker *et al* (2004) |
| 16s rDNAR | TCCTTAGAGTGCCCACCCG |  |  |
| RAPD primer | AGGAAGGTGC | RAPD analysis  Gene sequencing for MLST analysis | Mahenthiralingam et al (1996) |
| *acsA*-SF | GCCACACCTACATCGTCTAT | Gene sequencing for MLST analysis | Curran *et al* (2004) |
| *acsA*-SR | AGGTTGCCGAGGTTGTCCAC |  |  |
| *aroE*-SF | ATGTCACCGTGCCGTTCAAG |  |  |
| *aroE*-SR | TGAAGGCAGTCGGTTCCTTG |  |  |
| *guaA*-SF | AGGTCGGTTCCTCCAAGGTC |  |  |
| *guaA*-SR | GACGTTGTGGTGCGACTTGA |  |  |
| *mutL*-SF | AGAAGACCGAGTTCGACCAT |  |  |
| *mutL*-SR | GGTGCCATAGAGGAAGTCAT |  |  |
| *nuoD*-SF | ACGGCGAGAACGAGGACTAC |  |  |
| *nuoD*-SR | TGGCGGTCGGTGAAGGTGAA |  |  |
| *ppsA*-SF | GGTGACGACGGCAAGCTGTA |  |  |
| *ppsA*-SR | GTATCGCCTTCGGCACAGGA |  |  |
| *trpE*-SF | TTCAACTTCGGCGACTTCCA |  |  |
| *trpE*-SR | GGTGTCCATGTTGCCGTTCC |  |  |
| *mexR*OEF | CTGGATCAACCACATTTACATTAGG | *mexR* overexpression | This study |
| *mexR*OER | AGAATGTTCTTAAATATCCTCAAGC |  |  |
| q*mexB*F | GGTGAAGAACTTCCTCAT | RT-qPCR  RT-qPCR | This study |
| q*mexB*R | TGTTGGAAACGATGTAGT |  |  |
| q*mexR*F | AGCTTATCGACGAACAAC | RT-qPCR  RT-qPCR | This study |
| q*mexR*R | GGTGATCAGTGCCTTGTC |  |  |
| *mexR*EMF | GGTAGTTCATTGGTTTGGCC | clone intergenic region between *mexA* and *mexR* | This study |
| *mexR*EMR | TAGCTCGATGGCCGGTTATC |  |  |
| *mexR*HF | CGGGATCCATGAACTACCCCGTGAATCCCG | *mexR* cloned into pET28a | This study |
| *mexR*HR | CCCTCGAGAATATCCTCAAGCGGTTGCGCG |  |  |
| *mexR*MF | CGGAATTCTGCGCAGCGGTGATTTCCTCAAC | *mexR* point mutation | This study |
| *mexR*MR | CGGGATCCACCAGCAGGGCCGGAACCAGTAC |  |  |
| *mexR*TF | ATAAGAATGCGGCCGCAATGAACTACCCCGTGAATCCCGACCTG | Bacterial two-hybrid assay | This study |
| *mexR*TR | CCCTCGAGAGAATGTTCTTAAATATCCTCAAGC |  |  |

a: F, forward; R, reverse; S, sequencing; OE, overexpression; q, RT-qPCR; EM, EMSA; H, His-tagged; M, mutation; T, two-hybrid.

Curran, B., Jonas, D., Grundmann, H., Pitt, T., and Dowson, C.G. (2004). Development of a multilocus sequence typing scheme for the opportunistic pathogen *Pseudomonas aeruginosa*. *J Clin Microbiol* 42(12)**,** 5644-5649. doi: 10.1128/jcm.42.12.5644-5649.2004.

Mahenthiralingam, E., Campbell, M.E., Foster, J., Lam, J.S., and Speert, D.P. (1996). Random amplified polymorphic DNA typing of *Pseudomonas aeruginosa* isolates recovered from patients with cystic fibrosis. *J Clin Microbiol* 34(5)**,** 1129-1135.

Spilker, T., Coenye, T., Vandamme, P., and LiPuma, J.J. (2004). PCR-based assay for differentiation of *Pseudomonas aeruginosa* from other *Pseudomonas* species recovered from cystic fibrosis patients. *J Clin Microbiol* 42(5)**,** 2074-2079.
